# Predictors of Anorexia Nervosa and Obsessive‐Compulsive Disorder Comorbidity and Order of Diagnosis in a Danish National Cohort

**DOI:** 10.1002/eat.24486

**Published:** 2025-06-17

**Authors:** Lisa Yujia Zhu, Janne Tidselbak Larsen, Judith Becker Nissen, James J. Crowley, Manuel Mattheisen, Cynthia M. Bulik, Liselotte Vogdrup Petersen, Zeynep Yilmaz

**Affiliations:** ^1^ Department of Psychology University of Western Ontario London Ontario Canada; ^2^ National Centre for Register‐Based Research Aarhus University Aarhus Denmark; ^3^ Department of Clinical Medicine Aarhus University Aarhus Denmark; ^4^ Department of Genetics University of North Carolina at Chapel Hill Chapel Hill North Carolina United States; ^5^ Department of Community Health and Epidemiology Dalhousie University Halifax Nova Scotia Canada; ^6^ Department of Medical Epidemiology and Biostatistics Karolinska Institutet Stockholm Sweden; ^7^ Department of Psychiatry University of North Carolina at Chapel Hill Chapel HIll North Carolina United States; ^8^ Department of Nutrition University of North Carolina at Chapel Hill Chapel Hill North Carolina United States; ^9^ Department of Biomedicine Aarhus University Aarhus Denmark

**Keywords:** biological influences, Cox regression, environmental influences, etiological pathways, longitudinal analysis, nationwide register‐based study, order of onset, protective factors, psychiatric comorbidity

## Abstract

**Objective:**

Anorexia nervosa (AN) and obsessive‐compulsive disorder (OCD) are highly comorbid; however, limited research has examined etiological pathways specific to individuals with AN developing OCD or individuals with OCD developing AN. This exploratory study aimed to identify factors influencing AN‐OCD comorbidity with a focus on the order of diagnosis.

**Method:**

Using Danish national registers, 6449 individuals with AN and 9352 individuals with OCD were examined to assess the risk of subsequent OCD and AN. Explored predictors included parental characteristics, birth characteristics, childhood adversity, autoimmune and autoinflammatory diseases, psychiatric disorders, and prescriptions. Hazard ratios (HR) were calculated using Cox regression. Parallel analyses were conducted for the risk of subsequent anxiety disorder to determine predictors unique to AN‐OCD comorbidity.

**Results:**

Among individuals with AN, high birth weight (HR = 3.06) was uniquely associated with increased risk of subsequent OCD. For individuals with OCD, a history of other eating disorders (HR = 7.47) was associated with elevated risk of developing AN, whereas anxiety disorders in first‐degree (HR = 0.32) and female first‐degree relatives (HR = 0.22) were uniquely protective against AN.

**Discussion:**

These exploratory findings suggest that distinct pathways may be involved in the order of onset for AN‐OCD comorbidity. Specifically, for individuals with AN who subsequently developed OCD, high birth weight appeared to increase risk, whereas for individuals with OCD who later developed AN, familial anxiety disorders seemed to play a protective role. Findings could inform early screening and prevention efforts for individuals with AN at high risk for OCD, and vice versa.

**Summary**

- This study suggests that people with anorexia nervosa who had a higher birth weight may be at increased risk of developing obsessive‐compulsive disorder, whereas a family history of anxiety disorders may help protect individuals with obsessive‐compulsive disorder from developing anorexia nervosa.
- Identifying these factors could improve early detection and prevention of obsessive‐compulsive disorder in people with anorexia nervosa, and vice versa.

## 1 Introduction

Anorexia nervosa (AN) is a serious psychiatric disorder characterized by extremely low weight, severe food restriction, and cognitive distortions related to weight and shape (American Psychiatric Association 2022). The lifetime prevalence of AN is up to 4%, disproportionately affecting females (van Eeden et al. 2021). AN is difficult to treat, may become chronic if not detected and treated early, and has disturbingly high morbidity and mortality (Fichter et al. 2017; van Hoeken and Hoek 2020). Obsessive‐compulsive disorder (OCD)—an idiopathic neuropsychiatric disorder characterized by recurrent, unwanted thoughts and/or repetitive behaviors—is one of the most common comorbid conditions with AN. Roughly 20% of individuals with AN report a lifetime history of OCD (Drakes et al. 2021; Hambleton et al. 2022; Mandelli et al. 2020), an estimate much higher than the general population prevalence of OCD (Fineberg et al. 2013). Conversely, individuals with OCD have a 17‐fold higher risk of receiving a comorbid diagnosis of AN compared with matched individuals without OCD from the general population (Cederlöf et al. 2015). While this high comorbidity may be partially attributable to Berkson's bias (i.e., where individuals diagnosed with one disorder are more likely to be diagnosed with a second) (Schwartzbaum et al. 2003), the reported single nucleotide polymorphism‐based genetic correlation between AN and OCD (*r*_g_ = 0.45) (Watson et al. 2019) suggests that shared etiological pathways may also contribute to the observed comorbidity. The overarching aim of this study was to assess etiological pathways associated with AN‐OCD comorbidity with a focus on the order of diagnosis.

The comorbidity of AN and OCD has been shown to affect symptom profiles, course of illness, and treatment outcomes for both disorders. For example, comorbid OCD may predict more negative outcomes for individuals with AN, and vice versa (Carrot et al. 2017). The presence of OCD or obsessive‐compulsive symptoms is associated with earlier AN onset, increased severity of AN symptoms, lower body mass index following discharge, as well as longer duration of illness (Błachno et al. 2014; Duffy et al. 2019; Kwok et al. 2020). Comorbid OCD symptoms are especially pronounced in the subpopulation of individuals with AN with compulsive physical exercise (Błachno et al. 2016; Naylor et al. 2011), also an established predictor of poorer clinical outcomes including greater eating disorder symptomatology (Noetel et al. 2016; Young et al. 2018), relapse (de Rijk et al. 2024), and higher energy requirements for weight gain (Marzola et al. 2013). AN comorbidity in individuals with OCD may lead to a significant lag time before response to selective serotonin reuptake inhibitors (Dell'Osso et al. 2007) and poor treatment adherence (Steinglass 2007). Given the impact of AN‐OCD comorbidity on illness severity and treatment response, early detection of individuals at risk is crucial. Identification of specific risk factors contributing to this comorbid pattern may facilitate targeted screening and prevention efforts, ultimately improving prognosis.

Large‐scale population studies using national register data allow for the careful examination of a wide range of potential risk factors while being mindful of temporality. This research design has made notable contributions to our understanding of the etiology of psychiatric disorders by identifying various predictors associated with AN and OCD (Yilmaz et al. 2022; Meier et al. 2015; Larsen et al. 2017). Previous register‐based studies have linked AN to birth characteristics (e.g., parental age at birth, gestational age, multiple birth, cesarean section delivery), autoimmune and autoinflammatory diseases, and severe infections requiring hospitalization (Breithaupt et al. 2019; Javaras et al. 2017; Larsen et al. 2020; Hedman et al. 2019; Mårild et al. 2017; Zerwas et al. 2017). Similarly, studies have associated increased OCD risk with parental characteristics (e.g., age, education, occupation, income), birth characteristics (e.g., gestational age, birth weight, cesarean section delivery, Apgar score, maternal smoking), early‐life family composition (e.g., birth order, number of siblings), and personal and familial history of other psychiatric disorders (Yilmaz et al. 2022; Brander et al. 2016).

Despite extensive research on individual risk factors for AN and OCD, no study to date has systematically examined the predictors that shape the development of OCD following AN or vice versa. This study addressed this gap using an exploratory approach, selecting predictors based on prior register‐based studies that have linked them to AN or OCD. Specifically, these included parental characteristics, birth characteristics, autoimmune and autoinflammatory diseases, and personal and familial history of other psychiatric disorders. As the development of a subsequent diagnosis may be influenced by different factors than those contributing to initial risk, we also examined variables not previously identified as significant risk factors for AN or OCD, such as age at initial diagnosis and childhood adversity (Larsen et al. 2017; Chatwin et al. 2023). Finally, we explored whether prescriptions for various psychiatric medications prior to and following the initial AN or OCD diagnosis were associated with the development of the subsequent diagnosis.

To distinguish predictors uniquely influencing AN‐OCD comorbidity from those associated with broader psychiatric comorbidity, we conducted parallel analyses examining the risk of developing any anxiety disorder among individuals with an initial diagnosis of AN or OCD. Anxiety disorders were chosen as a comparison due to their frequent co‐occurrence with both AN and OCD (Sharma et al. 2021; Catone 2021).

## 2 Method

### 2.1 Data Sources

This study was conducted using information from nationwide Danish registers. All Danish citizens are assigned a unique identification number which enables linkage across various national registers. The Danish Civil Registration System contains data on all Danish citizens since 1968 and includes information on date and place of birth, identity of parents, and continuously updated residential information and vital status (Pedersen 2011). The Psychiatric Central Research Register (Mors et al. 2011) and the National Patient Register (Lynge et al. 2011) have recorded all inpatient contacts to Danish psychiatric and general hospitals since 1969 and 1977, respectively, and include information on admission and discharge dates and diagnoses using International Classification of Diseases (ICD)‐8 or, since 1994, ICD‐10 diagnostic codes. Outpatient contacts have been recorded since 1995. The Medical Birth Register (Bliddal et al. 2018) was established in 1973 and contains information on birth characteristics such as birth weight, gestational age, birth complications, and maternal pregnancy conditions. All prescription medications sold at pharmacies since 1994 have been recorded in the National Prescription Registry (Kildemoes et al. 2011), which includes date of prescription redemption, indication for prescription, and Anatomical Therapeutic Chemical (ATC) codes. Information regarding primary occupation and yearly income is recorded in the Integrated Database for Labour Market Research (Petersson et al. 2011), established in 1980. Finally, the Population's Education Register (Jensen and Rasmussen 2011) has recorded the highest completed education for each individual since 1981.

### 2.2 Study Population

**FIGURE 1:**
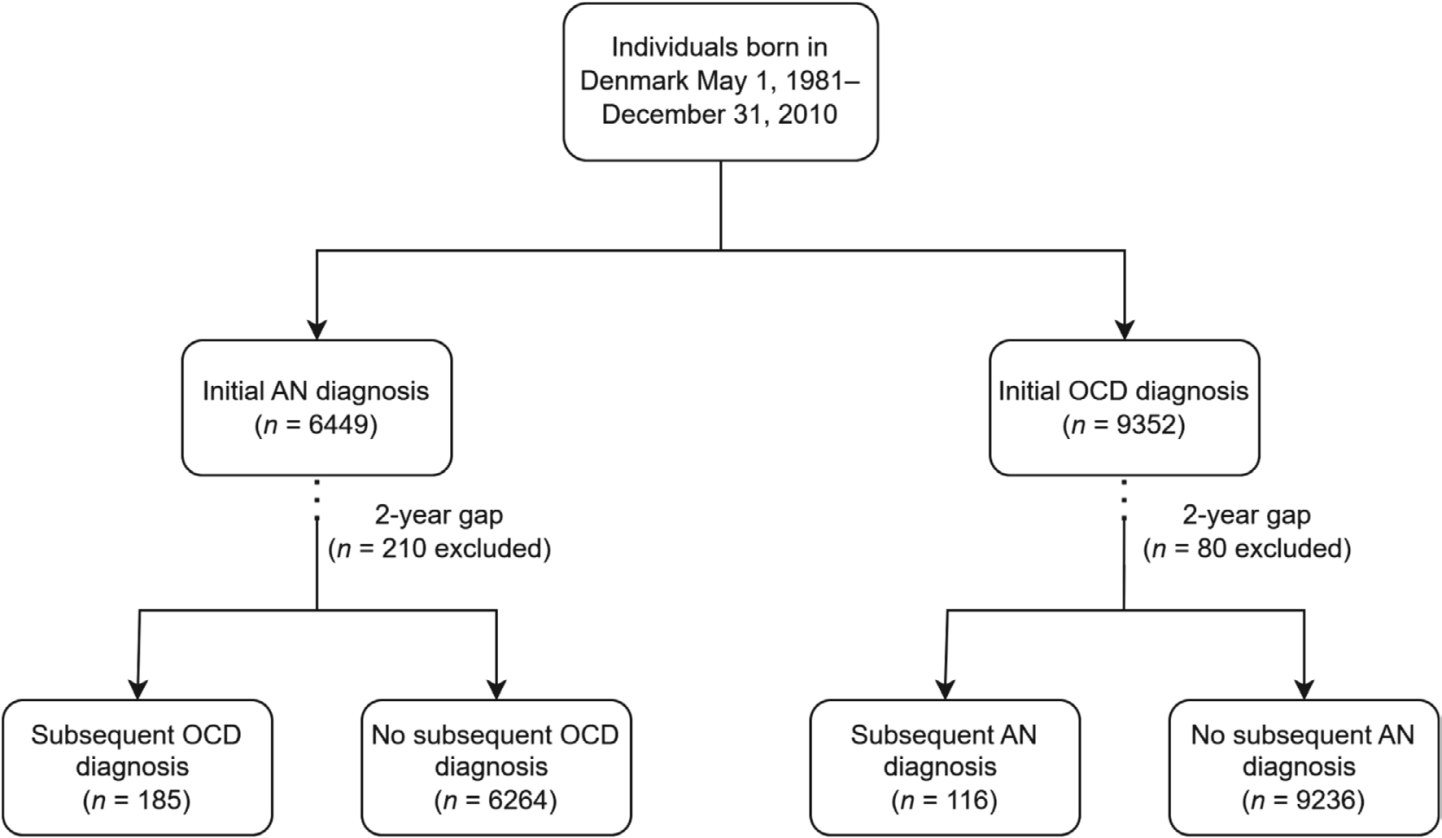
Flowchart of study sample for AN‐OCD analyses. *Note*: Individuals were excluded if their subsequent disorder occurred within 2 years of the initial disorder. Parallel follow‐up of the study population was conducted for subsequent anxiety disorder, to identify unique risk factors for AN‐OCD comorbidity, rather than risk for nonspecific psychiatric comorbidity. AN = anorexia nervosa; OCD = obsessive‐compulsive disorder.

The study population included individuals born in Denmark between May 1, 1981 and December 31, 2010 who were diagnosed with AN or OCD in the Psychiatric Central Research Register or the National Patient Register. Onset was defined as the date of the first recorded in‐ or outpatient hospital contact leading to diagnosis. Only AN diagnoses occurring after age six were included because it is unlikely to have a reliable diagnosis of AN prior to that age and earlier diagnoses were likely to be coding errors. Index individuals were followed from 2 years after the onset of the initial disorder—or if preceded by the onset, from their 6th birthday—and until the onset of the subsequent disorder (OCD or AN), death, emigration, or December 31, 2016, whichever came first (see Figure 1 for flowchart). This two‐year gap was arbitrarily chosen to limit ambiguity regarding the order of onset. Consequently, individuals were excluded if their subsequent disorder occurred within 2 years of the initial disorder (*n* = 210 for AN with subsequent OCD; *n* = 80 for OCD with subsequent AN). First‐degree relatives (i.e., parents, full siblings, and children) were identified using information from the Civil Registration System.

### 2.3 Explored Predictors

We explored the following predictors in relation to the risk of developing a subsequent diagnosis of OCD or AN. The same predictors were examined in the parallel analyses for subsequent anxiety disorder.

#### 2.3.1 Parental Characteristics

Maternal and paternal socio‐economic status was evaluated in the year preceding the birth of the individual with AN or OCD to avoid influence from the individual's psychiatric disorder(s) on socio‐economic status. Educational level was defined as the highest completed education at that time (high school or above, less than high school). Occupational level was defined as employment or unemployment, which included unemployed, on sick pay, pensioner, undergoing education, and other. Income was dichotomized as low (lowest tertile) versus medium to high, based on population distributions stratified by sex and calendar year.

#### 2.3.2 Birth Characteristics

Based on information from the Danish Medical Birth Registry, multiple birth (twin, triplet, quadruplet), gestational age (pre‐term [< 37 weeks], term, post‐term [> 41 weeks]), delivery by cesarean section, birth weight (< 2500 g, 2500–4499 g, ≥ 4500 g), and self‐reported maternal smoking during pregnancy were tested as predictors.

#### 2.3.3 Adversity Score

The following childhood adversities were assessed: familial death (death of a parent or sibling), family disruption (the child not sharing an address with both parents), parental disability (at least one parent receiving disability pension), parental general medical illness (at least one parent diagnosed in a hospital with any of the 19 diagnosis included in the Charlson Comorbidity Index, diagnostic codes as specified by Raedkjaer et al. (2018) excluding dementia), parental psychiatric illness (at least one parent diagnosed in hospital with any psychiatric disorder excluding substance use disorder), and parental substance use (at least one parent diagnosed in hospital with substance use disorder). Due to the small number of cases exposed to each individual domain of adversity, analyzing them separately would have led to insufficient power. To address this, we followed the methodology of a previous study on childhood adversity and risk of eating disorders (Larsen et al. 2017) and constructed an adversity score by counting the number of different adversities experienced at least once during the first 6 years of life. The adversity score was categorized as 0, 1, or 2 or more.

#### 2.3.4 Autoimmune and Autoinflammatory Diseases

Personal history of autoimmune and autoinflammatory diseases was assessed based on in‐ or outpatient hospital contacts recorded in the National Patient Register that resulted in a diagnosis of at least one of 61 diseases. Diseases were grouped into autoimmune diseases with suspected presence of brain‐reactive antibodies, other autoimmune or autoinflammatory diseases, and any autoimmune or autoinflammatory diseases. This definition is consistent with a previous study (Zerwas et al. 2017), where diagnostic codes and categories are specified.

#### 2.3.5 Psychiatric Disorders

Age at the initial diagnosis (per year and per SD) was examined in association with the risk of developing subsequent OCD or AN. Personal history of other eating disorders and other psychiatric disorders was also examined as potential predictors. These were treated as time‐dependent variables, meaning individuals could be diagnosed with them at any point before the onset of the subsequent disorder. Additionally, psychiatric disorders in first‐degree relatives—including OCD, any eating disorder, any anxiety disorder, and any psychiatric disorder—were also analyzed as time‐dependent predictors. Anxiety disorders in relatives were examined both collectively and separately by sex, as previous research suggests sex‐specific familial anxiety disorders may be linked to differing risks for eating disorders (Meier et al. 2015). Diagnostic codes are listed in Table 1.

**TABLE 1:** International classification of diseases (ICD) diagnostic codes for included disorders.

Disorder	ICD‐8 codes	ICD‐10 codes
Index individuals		
Anorexia nervosa	306.50	F50.0, F50.1
Obsessive‐compulsive disorder	300.39	F42
Other eating disorders	306.58, 306.59	F50.2, F50.3, F50.8, F50.9
Other psychiatric disorders	290–315 (excluding 306.50 and 300.39)	F00‐F99 (excluding F50.0, F50.1, and F42)
Anxiety disorder (excluding obsessive‐compulsive disorder)	300.x9 (excluding 300.39 and 300.49), 305.x9, 305.68, 307.99	F40–F48 (excluding F42)
First‐degree relatives		
Any eating disorder	306.5x	F50.0, F50.1, F50.2, F50.3, F50.8, F50.9
Any psychiatric disorder	290–315	F00–F99
Anxiety disorder	300.x9, 305.x9, 305.68, 307.99	F40–F48

#### 2.3.6 Prescriptions

Prescriptions were defined as at least one redeemed prescription recorded in the Danish National Prescription Registry for antidepressants, anxiolytics, hypnotics and sedatives, or other medications. The specific prescriptions included are listed in Table S1. The broad category of other medications was examined because previous research has observed increased use of a wide range of medications among individuals with AN, none being specific to AN treatment (Clausen et al. 2023). Prescriptions were categorized as occurring prior to or following the initial diagnosis based on the date of the first redeemed prescription. Additionally, prescriptions with OCD or anxiety indication—that is, those with a note in the registry stating that they were specifically for treating those disorders—were also included as predictors, allowing for a more precise investigation of how targeted medications may shape AN‐OCD comorbidity risk.

### 2.4 Statistical Analysis

Demographic characteristics, including age at subsequent diagnosis, sex, and origin of parents were compared between individuals with AN and individuals with OCD using an independent samples *t*‐test and chi‐squared tests. Cox proportional hazards regression was used to estimate the risk of developing the subsequent disorder (OCD or AN) based on each predictor. Autoimmune and autoinflammatory diseases, psychiatric disorders, and prescriptions following initial diagnosis were treated as time‐dependent variables, as they could plausibly emerge before the initial diagnosis or during the time period between the initial and subsequent diagnoses, potentially influencing the development of the subsequent diagnosis. All other predictors were treated as time‐independent, meaning they were assessed only if present prior to the initial diagnosis, based on the assumption that they reflect stable or early‐life characteristics. Analyses were adjusted for age as the underlying time scale, time‐dependent calendar years (categorized as 1987–1992, 1993–1994, 1995–1999, 2000–2004, 2005–2009, 2010–2012, 2013–2016), and sex using separate underlying baseline hazards. To identify predictors unique to AN‐OCD comorbidity, rather than nonspecific psychiatric comorbidity, we also estimated the risk of any subsequent anxiety disorder in individuals with initial AN or OCD. Additional analyses were conducted adjusted for personal history of any psychiatric disorders except AN and OCD prior to the initial diagnosis. This adjustment ensured that associations between prescriptions prior to initial diagnosis and risk for the subsequent disorder were not confounded by pre‐existing psychiatric disorders. Model assumptions were evaluated graphically using log–log survival plots, and results are presented as hazard ratios (HRs) with 95% confidence intervals. The Benjamini–Hochberg procedure was used to control for multiple testing in all analyses.

## 3 Results

Of all the individuals included in the registers, 6449 (0.44%) were diagnosed with AN and 9352 (0.67%) with OCD, corresponding to incidence rates of 22 per 100,000 person‐years for AN and 33 per 100,000 person‐years for OCD. These index individuals were followed for a combined total of 92,855.81 person‐years. One hundred and eighty‐five (2.87%) individuals with AN developed subsequent OCD and 116 (1.24%) individuals with OCD developed subsequent AN (see Figure 1 for flowchart). Demographic characteristics and results of the independent samples *t*‐test and chi‐squared tests are presented in Table 2. Individuals with AN were diagnosed with subsequent OCD at a significantly older age than individuals with OCD who developed AN. In terms of sex distribution, the proportion of females was significantly higher among individuals with AN compared to individuals with OCD. No significant differences were observed for the origin of parents.

**TABLE 2:** Demographic characteristics of individuals with anorexia nervosa and obsessive‐compulsive disorder.

	Individuals with AN			Individuals with OCD			*p* ^a^
	*N* (%)	*n* subsequent OCD (%)	*M* (SD)	*N* (%)	*n* subsequent AN (%)	*M* (SD)	
Total	6449	185 (2.87)		9352	116 (1.24)		
Sex							0.036
Female	6010 (93.19)	174 (94.05)		5426 (58.02)	101 (87.07)		
Male	439 (6.81)	11 (5.95)		3926 (41.98)	15 (12.93)		
Origin of parents							0.101
Both parents born in Denmark	5748 (89.13)	174 (94.05)		8300 (88.75)	103 (88.79)		
One parent born in Denmark	543 (8.42)	11 (5.95)		777 (8.31)	13 (11.21)^b^		
Both parents born abroad	158 (2.45)	0 (0)		275 (2.94)			
Age at initial diagnosis			17.36 (4.20)			17.04 (6.18)	
Age at subsequent diagnosis			22.85 (4.60)			19.34 (4.64)	< 0.001
Duration between diagnoses (years)			6.28 (3.61)			5.35 (2.69)	0.011

*Note*: AN = anorexia nervosa; OCD = obsessive‐compulsive disorder.

^a^ Differences in demographic characteristics between individuals with AN vs. individuals with OCD were examined. An independent samples *t*‐test was conducted for age at subsequent diagnosis, while a Welch's *t*‐test was conducted for duration between diagnoses. Chi‐squared tests were conducted for sex and origin of parents.

^b^ Too few cases to report separately.

Hazard ratios (HRs) of subsequent OCD and AN risk are presented in Table 3, organized as significant predictors unique to subsequent OCD or AN, significant predictors also associated with subsequent anxiety disorder, and non‐significant predictors. Parallel demographic characteristics and hazard ratios for subsequent anxiety disorder are presented in Tables S2 and S3. See Tables S4 and S5 for the hazard ratios of additional analyses with adjustment for history of psychiatric disorders.

**TABLE 3:** Hazard ratios for subsequent OCD among individuals with AN and subsequent AN among individuals with OCD.

Predictor		Subsequent OCD among individuals with AN			Subsequent AN among individuals with OCD		
		Cases	HR (95% CI)	Adjusted *p*	Cases	HR (95% CI)	Adjusted *p*
Significant predictors unique to subsequent OCD or AN							
Birth weight	< 2500 g	10	0.85 (0.45–1.61)	0.810			
	2500–4499 g	165	1.00 (ref)				
	≥ 4500 g	10	**3.06 (1.61–5.81)**	< 0.05			
Other eating disorders	No	109	1.00 (ref)		91	1.00 (ref)	
	Yes	76	1.49 (1.11–2.00)	0.247	25	**7.47 (4.72–11.83)**	< 0.05
Significant predictors also associated with subsequent anxiety disorder							
Other psychiatric disorders	No	40	1.00 (ref)		41	1.00 (ref)	
	Yes	145	**2.41 (1.69–3.43)**	< 0.05	75	1.10 (0.75–1.62)	0.976
Any anxiety disorder in first‐degree relatives^a^	No	146	1.00 (ref)		105	1.00 (ref)	
	Yes	39	1.17 (0.82–1.67)	0.810	11	**0.32 (0.17–0.61)**	< 0.05
Any anxiety disorder in female first‐degree relatives^a^	No	157	1.00 (ref)		111	1.00 (ref)	
	Yes	28	1.19 (0.80–1.78)	0.810	5	**0.22 (0.09–0.54)**	< 0.05
Antidepressants following initial diagnosis	No	45	1.00 (ref)		39	1.00 (ref)	
	Yes	140	**4.43 (3.13–6.26)**	< 0.05	77	1.64 (1.09–2.48)	0.522
Antidepressants with OCD or anxiety indication following initial diagnosis	No	133	1.00 (ref)		65	1.00 (ref)	
	Yes	52	**3.75 (2.69–5.22)**	< 0.05	51	**1.91 (1.29–2.81)**	< 0.05
Anxiolytics following initial diagnosis	No	151	1.00 (ref)		100	1.00 (ref)	
	Yes	34	**2.15 (1.46–3.16)**	< 0.05	16	1.71 (0.98–2.98)	0.976
Other medications following initial diagnosis	No	144	1.00 (ref)		98	1.00 (ref)	
	Yes	41	**1.90 (1.34–2.70)**	< 0.05	18	0.79 (0.48–1.32)	0.976
Any medications following initial diagnosis	No	38	1.00 (ref)		37	1.00 (ref)	
	Yes	147	**4.47 (3.10–6.44)**	< 0.05	79	1.53 (1.01–2.32)	0.976
Any medications with OCD or anxiety indication following initial diagnosis	No	128	1.00 (ref)		65	1.00 (ref)	
	Yes	57	**3.85 (2.78–5.33)**	< 0.05	51	1.85 (1.26–2.74)	0.061
Non‐significant predictors							
Age at initial diagnosis (per year)			1.05 (1.00–1.11)	0.810		1.04 (0.97–1.11)	0.976
Age at initial diagnosis (per SD)			1.25 (1.01–1.55)	0.810		1.26 (0.83–1.92)	0.976
Maternal education	High school or above	117	1.00 (ref)		84	1.00 (ref)	
	Less than high school	65	1.14 (0.83–1.54)	0.810	27	0.69 (0.44–1.07)	0.976
Paternal education	High school or above	133	1.00 (ref)		94	1.00 (ref)	
	Less than high school	47	1.08 (0.77–1.50)	0.810	16	0.52 (0.30–0.88)	0.449
Maternal unemployment	No	157	1.00 (ref)		92	1.00 (ref)	
	Yes	27	0.89 (0.59–1.35)	0.810	24	1.01 (0.64–1.59)	0.976
Paternal unemployment	No	170	1.00 (ref)		105	1.00 (ref)	
	Yes	14	0.83 (0.48–1.44)	0.810	11	0.80 (0.43–1.49)	0.976
Maternal income	Medium to high	168	1.00 (ref)		106	1.00 (ref)	
	Low	16	0.85 (0.51–1.42)	0.810	10	0.96 (0.50–1.85)	0.976
Paternal income	Medium to high	170	1.00 (ref)		107	1.00 (ref)	
	Low	14	0.80 (0.46–1.38)	0.810	9	0.98 (0.50–1.95)	0.976
Multiple birth^b^	Singleton						
	Twin, triplet or quadruplet						
Gestational age	Preterm	7	0.60 (0.28–1.27)	0.810	5	0.59 (0.24–1.46)	0.976
	Term	162	1.00 (ref)		102	1.00 (ref)	
	Post‐term	14	0.86 (0.50–1.49)	0.810	8	0.81 (0.39–1.67)	0.976
Cesarean section	No	170	1.00 (ref)		100	1.00 (ref)	
	Yes	15	1.21 (0.71–2.07)	0.810	16	1.38 (0.81–2.38)	0.976
Smoking during pregnancy	No	46	1.00 (ref)		57	1.00 (ref)	
	Yes	20	1.30 (0.77–2.20)	0.810	13	0.59 (0.32–1.08)	0.976
Adversity score	0	141	1.00 (ref)		81	1.00 (ref)	
	1	36	0.78 (0.54–1.12)	0.810	29	0.80 (0.52–1.23)	0.976
	2 or more	8	0.80 (0.39–1.64)	0.810	6	0.66 (0.29–1.51)	0.976
Autoimmune diseases with brain‐reactive antibodies	No	176	1.00 (ref)				
	Yes	9	2.41 (1.23–4.72)	0.300			
Other autoimmune or autoinflammatory diseases	No	173	1.00 (ref)		110	1.00 (ref)	
	Yes	12	1.15 (0.64–2.08)	0.810	6	1.33 (0.58–3.04)	0.976
Any autoimmune or autoinflammatory disease	No	165	1.00 (ref)		110	1.00 (ref)	
	Yes	20	1.61 (1.01–2.57)	0.810	6	1.06 (0.47–2.43)	0.976
OCD in first‐degree relatives	No	179	1.00 (ref)				
	Yes	6	2.05 (0.91–4.64)	0.810			
Any eating disorder in first‐degree relatives	No	177	1.00 (ref)				
	Yes	8	1.09 (0.54–2.22)	0.810			
Any anxiety disorder in male first‐degree relatives	No	169	1.00 (ref)		110	1.00 (ref)	
	Yes	16	1.25 (0.75–2.10)	0.810	6	0.51 (0.22–1.15)	0.976
Any psychiatric disorder in first‐degree relatives	No	109	1.00 (ref)		71	1.00 (ref)	
	Yes	76	1.15 (0.85–1.54)	0.810	45	0.86 (0.59–1.26)	0.976
Antidepressants prior to initial diagnosis	No	150	1.00 (ref)		84	1.00 (ref)	
	Yes	35	1.62 (1.11–2.37)	0.377	32	1.51 (0.96–2.36)	0.976
Antidepressants with OCD or anxiety indication prior to initial diagnosis	No	178	1.00 (ref)		104	1.00 (ref)	
	Yes	7	2.48 (1.15–5.31)	0.540	12	1.55 (0.83–2.89)	0.976
Anxiolytics prior to initial diagnosis	No	178	1.00 (ref)		106	1.00 (ref)	
	Yes	7	1.16 (0.54–2.48)	0.810	10	1.35 (0.70–2.61)	0.976
Hypnotics and sedatives prior to initial diagnosis	No	175	1.00 (ref)				
	Yes	10	2.26 (1.18–4.33)	0.391			
Other medications prior to initial diagnosis	No				111	1.00 (ref)	
	Yes				5	0.79 (0.32–1.95)	0.976
Any medications prior to initial diagnosis	No	143	1.00 (ref)		81	1.00 (ref)	
	Yes	42	1.62 (1.14–2.31)	0.247	35	1.24 (0.81–1.90)	0.976
Any medications with OCD or anxiety indication prior to initial diagnosis	No	178	1.00 (ref)		104	1.00 (ref)	
	Yes	7	2.29 (1.07–4.91)	0.810	12	1.47 (0.79–2.74)	0.976
Hypnotics and sedatives following initial diagnosis	No	155	1.00 (ref)		106	1.00 (ref)	
	Yes	30	1.50 (1.00–2.26)	0.810	10	1.68 (0.85–3.31)	0.976

*Note*: Significant hazard ratios are bolded. Multiple testing was controlled for using the Benjamini–Hochberg procedure.

Abbreviations: AN = anorexia nervosa; OCD = obsessive‐compulsive disorder; ref. = reference level.

^a^ Although this predictor was also significant for subsequent anxiety disorder, it was associated with increased risk, opposite to the direction observed for subsequent AN.

^b^ Too few cases to report.

For individuals with AN, ≥ 4500 g birth weight (HR = 3.06), other psychiatric disorders (HR = 2.41), and most prescriptions (antidepressants [HR = 4.43], antidepressants with OCD or anxiety indication [HR = 3.75], anxiolytics [HR = 2.15], other medications [HR = 1.90], any medications [HR = 4.47], any medications with OCD or anxiety indication [HR = 3.85]) following initial diagnosis were significantly associated with increased risk of subsequent OCD. For individuals with OCD, other eating disorders (HR = 7.47) and prescription of antidepressants with OCD or anxiety indication following initial diagnosis (HR = 1.91) were significantly associated with increased risk of subsequent AN. Anxiety disorders in first‐degree relatives (HR = 0.32) and anxiety disorders in female first‐degree relatives (HR = 0.22) were significantly associated with decreased risk of subsequent AN.

To determine predictors unique to AN‐OCD comorbidity, rather than nonspecific psychiatric comorbidity, we conducted parallel analyses with subsequent anxiety disorder (see Table S3). For individuals with AN, all of the significant predictors for subsequent OCD were also significant for subsequent anxiety disorder, except for ≥ 4500 g birth weight. For individuals with OCD, all of the predictors significantly associated with increased risk of subsequent AN were also significant for subsequent anxiety disorder, except for other eating disorders. Interestingly, the predictors that were significantly associated with decreased risk of subsequent AN (i.e., anxiety disorders in first‐degree relatives, anxiety disorders in female first‐degree relatives) were significantly associated with increased risk of subsequent anxiety disorder.

## 4 Discussion

Using a nationwide, register‐based cohort, this exploratory study identified several factors shaping risk of AN‐OCD comorbidity that were specific to the order of diagnosis and unique from general psychiatric comorbidity. For individuals with AN, ≥ 4500 g birth weight was significantly associated with increased risk of subsequent OCD (HR = 3.06), while among individuals with OCD, a history of other eating disorders was the strongest predictor of subsequent AN (HR = 7.47). In contrast, a history of any anxiety disorders in first‐degree (HR = 0.32) and female first‐degree relatives (HR = 0.22) was associated with a decreased risk of subsequent AN among individuals with OCD.

The observed hazard ratios indicate meaningful effect sizes, particularly for high birth weight. Individuals with AN who had high birth weight had a threefold increase in risk of subsequent OCD, aligning with previous research indicating that high birth weight is associated with an increased risk for OCD in the general population (Brander et al. 2016). Among individuals with AN, high birth weight may amplify the shared genetic vulnerability to both AN and OCD (Watson et al. 2019). Further research is warranted to elucidate the interacting mechanisms underlying the association between high birth weight and AN‐OCD comorbidity, such as metabolic dysregulation, alterations in neurodevelopment, and maternal health (Rossi et al. 2013).

Similarly, the sevenfold increase in risk of subsequent AN among individuals with OCD who had a history of other eating disorders underscores the well‐documented rates of diagnostic transition within eating disorders (Ackard et al. 2011). This finding suggests that OCD could potentially serve as an intermediary step in the progression of other eating disorders toward AN, highlighting the importance of early identification and intervention in individuals with OCD who exhibit disordered eating.

Conversely, the inverse relationship between familial anxiety disorders and subsequent AN among individuals with OCD presents a novel and unexpected finding. The magnitude of the protective effect was substantial—individuals with OCD who had a first‐degree relative with an anxiety disorder had a 68% reduced risk of developing AN compared to those without familial anxiety. The protective effect was even stronger when restricting the analysis to female first‐degree relatives, with a 78% lower risk of subsequent AN. These results contrast with a previous Danish population register study, which linked both personal and familial anxiety disorders to an elevated risk of AN (Meier et al. 2015). A possible explanation is that individuals with OCD and familial anxiety may be more likely to develop an anxiety disorder instead of transitioning to AN, given that anxiety disorders tend to co‐aggregate within families (Shimada‐Sugimoto et al. 2015). This pattern may reflect higher polygenic risk scores for OCD and anxiety disorders among individuals with OCD and familial anxiety. Such genetic predispositions could reduce the likelihood of transitioning to AN, as they may outweigh genetic liability for AN in these individuals. Although the negative association between familial anxiety disorders and AN remained significant even after adjusting for personal history of other psychiatric diagnoses prior to OCD, it is plausible that many individuals developed comorbid anxiety disorders following their OCD diagnosis. Indeed, we found that familial anxiety disorders were significantly associated with an increased risk of subsequent anxiety disorder among individuals with OCD.

Further, individuals with comorbid OCD and anxiety disorders, particularly panic disorder and generalized anxiety disorder, are more likely to have visited a mental health center in the past 3 months, received psychotherapy, and taken psychiatric medication (García‐Soriano et al. 2014). Such psychological and pharmacological treatments may help prevent the progression of OCD to AN by effectively managing obsessions and compulsions before the emergence of dysfunctional attitudes and behaviors involving food, eating, and weight. Moreover, individuals with familial anxiety disorders may be more adept at recognizing their OCD symptoms early and understanding their treatment needs. This heightened awareness may further promote treatment seeking and adherence, mitigating the risk of subsequent AN. Future research should investigate whether treatment adherence for OCD acts as a protective factor against the development of AN.

## 5 Strengths and Limitations

Using register data allows for the analysis of large sample sizes, along with comprehensive sociodemographic and clinical information on individuals with AN or OCD and their relatives, collected over decades. Throughout the time period examined in this study, the definition of AN remained consistent, despite changes to the specific ICD codes (306.50 in ICD‐8 to F50.0 and F50.1 in ICD‐10), which were accounted for in this study. Register data also circumvents selection and recall bias, as well as inconsistencies related to determining order of onset—whether it be onset of symptoms, meeting full diagnostic criteria, or receiving a professional diagnosis—which often accompany self‐report. Notably, the positive predictive values for AN and OCD in Danish registers are high (Egedal et al. 2024; Nissen et al. 2017).

However, register‐based studies also have limitations. Coding errors could potentially occur, and symptom overlap between psychiatric disorders—particularly among closely related conditions such as AN, OCD, and anxiety disorders—can lead to misdiagnosis, possibly impacting the accuracy of our findings. Additionally, reliance on registers may have led to incomplete capture of prescriptions relevant to OCD or anxiety. Some medications are prescribed off‐label in Denmark and may not have been recorded with the intended indication, limiting our ability to assess the precise role of prescriptions with OCD or anxiety indication in AN‐OCD comorbidity.

Registers also have lower sensitivity compared to self‐report methods, as they only include individuals who received an in‐ or outpatient hospital diagnosis, which may not capture all cases. In our study, the incidence rates were 22 per 100,000 person‐years for AN and 33 per 100,000 person‐years for OCD, which are comparable to those observed in other register‐based studies (van Eeden et al. 2021; Rintala et al. 2017), but considerably lower than estimates from population‐based research (Fineberg et al. 2013; Silén et al. 2020). These lower rates likely reflect the low levels of help‐seeking associated with psychiatric disorders, particularly AN (Hart et al. 2011). Consequently, our results likely underestimate the true rates of AN‐OCD comorbidity and are specific to individuals who are more likely to seek care from hospitals. However, if help‐seeking was the only driver of our findings, we would expect similar patterns across the subsequent OCD or AN and subsequent anxiety disorder analyses. Instead, several predictors that were significant for subsequent OCD or AN were either not significant for subsequent anxiety disorder or had opposite effects, suggesting that these factors shape actual disorder risk rather than merely reflecting healthcare utilization. It is also worth noting that the sensitivity is likely minimally impacted by structural barriers, as private healthcare is relatively uncommon in Denmark and access to care is generally equitable (Gundgaard 2006).

Register‐based designs also introduce the potential for Berkson's bias (Schwartzbaum et al. 2003), which may inflate observed comorbidity rates and limit the generalizability of our findings to the broader community. To mitigate this, we examined two cohorts of individuals with initial diagnoses and tracked them for the development of the subsequent disorders, beginning 2 years after the initial diagnoses. This two‐year gap reduced the likelihood that the observed comorbidity was solely due to heightened clinical attention. It also helped distinguish truly separate diagnoses, particularly among individuals with OCD and prior eating disorders, reducing the risk that prior eating disorders simply reflect the early stages of AN. However, this approach excluded individuals diagnosed with the subsequent disorder shortly after the initial disorder, who may have different etiological pathways compared to those included in our study. Despite this limitation, our study successfully captured an adequate sample size, ensuring the robustness of our results.

In summary, this exploratory study investigated factors influencing the risk of AN‐OCD comorbidity with a focus on the order of diagnosis. We identified that high birth weight increased the risk of subsequent OCD among individuals with AN, whereas a history of other eating disorders elevated the risk of subsequent AN among those with OCD. Conversely, familial anxiety disorders were uniquely associated with a decreased risk of subsequent AN among individuals with OCD. These results suggest that distinct pathways may contribute to AN‐OCD comorbidity depending on the order of onset. As this is a first step in understanding these pathways, further research is warranted to replicate and extend these findings. Future studies could leverage genetic analyses, such as polygenic risk scores, to identify genetic predispositions that influence the order in which these disorders manifest. For instance, individuals with higher polygenic risk scores for AN may be more likely to develop AN first, followed by OCD. Additionally, studies could explore how demographic characteristics—such as age at each diagnosis and sex—along with gene–environment interactions influence the progression from one disorder to another. Such research could inform early screening and prevention efforts tailored to individuals with specific risk profiles for AN with subsequent OCD or OCD with subsequent AN.

## Author Contributions

**Lisa Yujia Zhu:** writing – original draft. **Janne Tidselbak Larsen:** formal analysis, methodology. **Judith Becker Nissen:** methodology. **James J. Crowley:** writing – review and editing. **Manuel Mattheisen:** conceptualization. **Cynthia M. Bulik:** funding acquisition, writing – review and editing. **Liselotte Vogdrup Petersen:** methodology, supervision, writing – review and editing. **Zeynep Yilmaz:** conceptualization, funding acquisition, methodology, supervision, writing – review and editing.

## Ethics Statement

This study was approved by the Danish Data Protection Agency, and data access was approved by Statistics Denmark and the Danish Health Data Authority.

## Conflicts of Interest

The authors declare no conflicts of interest.

## Supporting information


**Data S1.** Supporting Information.

## Data Availability

Access to individual level Danish data are governed by Danish authorities and can only be granted with prior approval. The governing bodies include the Danish Data Protection Agency and the Danish Health Data Authority.
