# Supplementary material for: Predictors of Anorexia Nervosa and Obsessive‐Compulsive Disorder Comorbidity and Order of Diagnosis in a Danish National Cohort
Source: Int J Eat Disord. 2025 Jun 17;58(9):1817–29. doi: 10.1002/eat.24486 (PMC12423569; doi:10.1002/eat.24486)
Supplement: Supplementary file 1 — Data S1. Supporting Information. [file EAT-58-1817-s001.docx]

**Supporting Information**

**Table 1**

*Included Prescriptions*

| Category | Medication | Anatomical Therapeutic Chemical (ATC) codes |
| --- | --- | --- |
| Antidepressants | Tricyclic antidepressants | N06AA |
|  | Selective serotonin reuptake inhibitors | N06AB |
|  | Monoamine oxidase A inhibitors | N06AF, N06AG |
|  | Noradrenergic and specific serotonergic antidepressants | N06AX03, N06AX11 |
|  | Serotonin and norepinephrine reuptake inhibitors | N06AX16, N06AX21 |
|  | Norepinephrine reuptake inhibitors | N06AX18 |
| Anxiolytics | Benzodiazepine derivatives | N05BA |
|  | Dibenzo-bicyclo-octadiane derivatives | N05BD |
|  | Azaspirodecanedione derivatives | N05BE |
| Hypnotics and sedatives | Benzodiazepine derivatives | N05CD |
|  | Benzodiazepine related medications | N05CF |
| Other medications | Haloperidol | N05AD01 |
|  | Sertindole | N05AE03 |
|  | Ziprasidone | N05AE04 |
|  | Pimozide | N05AG02 |
|  | Clozapine | N05AH02 |
|  | Olanzapine | N05AH03 |
|  | Quetiapine | N05AH04 |
|  | Sulpiride | N05AL01 |
|  | Risperidone | N05AX08 |
|  | Aripiprazole | N05AX12 |

**Table 2**

*Demographic Characteristics of Individuals with Anorexia Nervosa and Obsessive-Compulsive Disorder with Subsequent Anxiety Disorder*

|  | Individuals with AN | | | Individuals with OCD | | | *p*^a^ |
| --- | --- | --- | --- | --- | --- | --- | --- |
|  | *N* (%) | *n* subsequent anxiety disorder (%) | *M* (*SD*) | *N* (%) | *n* subsequent anxiety disorder (%) | *M* (*SD*) |  |
| Total | 5568 | 576 (10.34) |  | 6917 | 772 (!1.16) |  |  |
| Sex |  |  |  |  |  |  | < .001 |
| Female | 5141 (92.33) | 547 (94.97) |  | 3817 (55.18) | 514 (66.58) |  |  |
| Male | 427 (7.67) | 29 (5.03) |  | 3100 (44.82) | 258 (33.42) |  |  |
| Origin of parents |  |  |  |  |  |  | .651 |
| Both parents born in Denmark | 4980 (89.44) | 514 (89.24) |  | 6117 (88.43) | 689 (89.25) |  |  |
| One parent born in Denmark | 458 (8.23) | 53 (9.20) |  | 589 (8.52) | 66 (8.55) |  |  |
| Both parents born abroad | 130 (2.33) | 9 (1.56) |  | 211 (3.05) | 17 (2.20) |  |  |
| Age at initial diagnosis |  |  | 17.10 (4.10) |  |  | 15.93 (5.86) |  |
| Age at subsequent diagnosis |  |  | 23.08 (4.87) |  |  | 21.75 (5.53) | < .001 |

*Note*. AN = anorexia nervosa; OCD = obsessive-compulsive disorder.

^a^ Differences in demographic characteristics between individuals with AN vs. individuals with OCD were examined. An independent samples t-test was conducted for age at subsequent diagnosis, while chi-squared tests were conducted for sex and origin of parents.

^b^ Too few cases to report separately.

**Table 3**

*Hazard Ratios for Subsequent Anxiety Disorder Risk Among Individuals with AN and OCD*

| Predictor | | Subsequent anxiety disorder among individuals with AN | | | Subsequent anxiety disorder among individuals with OCD | | |
| --- | --- | --- | --- | --- | --- | --- | --- |
|  | | Cases | *HR* (95% CI) | Adjusted *p* | Cases | *HR* (95% CI) | Adjusted *p* |
| **Significant predictors unique to subsequent anxiety disorder** | |  |  |  |  |  |  |
| Age at initial diagnosis (per year) | |  | 1.04 (1.01-1.07) | .056 |  | **1.06 (1.04-1.08)** | <.05 |
| Age at initial diagnosis (per *SD*) | |  | 1.18 (1.06-1.33) | .056 |  | **1.40 (1.23-1.59)** | <.05 |
| Maternal education | High school or above | 348 | 1.00 (ref) |  | 468 | 1.00 (ref) |  |
|  | Less than high school | 212 | **1.38 (1.16-1.64)** | <.05 | 281 | 1.22 (1.05-1.41) | .231 |
| Paternal education | High school or above | 389 | 1.00 (ref) |  | 518 | 1.00 (ref) |  |
|  | Less than high school | 159 | **1.38 (1.15-1.66)** | <.05 | 214 | **1.29 (1.10-1.51)** | <.05 |
| Maternal income | Medium to high | 500 | 1.00 (ref) |  | 696 | 1.00 (ref) |  |
|  | Low | 76 | **1.50 (1.18-1.91)** | <.05 | 73 | 1.00 (0.79-1.28) | .975 |
| Birth weight | <2500g | 57 | **1.75 (1.33-2.31)** | <.05 | 47 | 0.99 (0.74-1.33) | .975 |
|  | 2500–4499g | 502 | 1.00 (ref) |  | 700 | 1.00 (ref) |  |
|  | ≥4500g | 15 | 1.50 (0.90-2.51) | .319 | 19 | 0.94 (0.60-1.49) | .975 |
| Adversity score | 0 | 399 | 1.00 (ref) |  | 488 | 1.00 (ref) |  |
|  | 1 | 139 | 1.20 (0.99-1.45) | .319 | 219 | 1.17 (1.00-1.37) | .975 |
|  | 2 or more | 38 | **1.70 (1.22-2.37)** | <.05 | 65 | **1.53 (1.18-1.99)** | <.05 |
| Other autoimmune or autoinflammatory diseases | No | 528 | 1.00 (ref) |  | 736 | 1.00 (ref) |  |
|  | Yes | 48 | **1.61 (1.20-2.17)** | <.05 | 36 | 1.28 (0.92-1.80) | .975 |
| Any autoimmune or autoinflammatory disease | No | 518 | 1.00 (ref) |  | 726 | 1.00 (ref) |  |
|  | Yes | 58 | **1.58 (1.20-2.08)** | <.05 | 46 | 1.30 (0.96-1.75) | .975 |
| Other eating disorders | No | 346 | 1.00 (ref) |  |  |  |  |
|  | Yes | 230 | **1.66 (1.40-1.96)** | <.05 |  |  |  |
| Any anxiety disorder in male first degree relatives | No | 522 | 1.00 (ref) |  | 671 | 1.00 (ref) |  |
|  | Yes | 54 | **1.58 (1.20-2.10)** | <.05 | 101 | **1.42 (1.15-1.76)** | <.05 |
| Any psychiatric disorder in first degree relatives | No | 315 | 1.00 (ref) |  | 381 | 1.00 (ref) |  |
|  | Yes | 261 | **1.58 (1.34-1.87)** | <.05 | 391 | **1.45 (1.25-1.67)** | <.05 |
| Antidepressants prior to initial diagnosis | No | 471 | 1.00 (ref) |  | 546 | 1.00 (ref) |  |
|  | Yes | 105 | **1.89 (1.52-2.35)** | <.05 | 226 | 1.24 (1.05-1.46) | .259 |
| Antidepressants with OCD or anxiety indication prior to initial diagnosis | No | 559 | 1.00 (ref) |  | 702 | 1.00 (ref) |  |
|  | Yes | 17 | **2.33 (1.43-3.79)** | <.05 | 70 | 1.19 (0.93-1.54) | .975 |
| Anxiolytics prior to initial diagnosis | No | 546 | 1.00 (ref) |  | 703 | 1.00 (ref) |  |
|  | Yes | 30 | **2.20 (1.52-3.18)** | <.05 | 69 | **1.48 (1.15-1.89)** | <.05 |
| Hypnotics and sedatives prior to initial diagnosis | No | 555 | 1.00 (ref) |  | 736 | 1.00 (ref) |  |
|  | Yes | 21 | **1.94 (1.24-3.01)** | <.05 | 36 | 1.66 (1.18-2.35) | .087 |
| Any medications prior to initial diagnosis | No | 453 | 1.00 (ref) |  | 495 | 1.00 (ref) |  |
|  | Yes | 123 | **1.86 (1.52-2.28)** | <.05 | 277 | **1.35 (1.15-1.57)** | <.05 |
| Any medications with OCD or anxiety indication prior to initial diagnosis | No | 558 | 1.00 (ref) |  | 698 | 1.00 (ref) |  |
|  | Yes | 18 | **2.38 (1.48-3.82)** | <.05 | 74 | 1.21 (0.95-1.55) | .975 |
| **Significant predictors also associated with subsequent OCD or AN** | |  |  |  |  |  |  |
| Other psychiatric disorders | No | 162 | 1.00 (ref) |  | 235 | 1.00 (ref) |  |
|  | Yes | 414 | **2.32 (1.93-2.78)** | <.05 | 537 | **1.77 (1.51-2.06)** | <.05 |
| Any anxiety disorder in first degree relatives^a^ | No | 443 | 1.00 (ref) |  | 537 | 1.00 (ref) |  |
|  | Yes | 133 | **1.54 (1.26-1.87)** | <.05 | 235 | **1.45 (1.24-1.69)** | <.05 |
| Any anxiety disorder in female first degree relatives^a^ | No | 489 | 1.00 (ref) |  | 611 | 1.00 (ref) |  |
|  | Yes | 87 | **1.41 (1.12-1.77)** | <.05 | 161 | **1.40 (1.18-1.67)** | <.05 |
| Antidepressants following initial diagnosis | No | 192 | 1.00 (ref) |  | 212 | 1.00 (ref) |  |
|  | Yes | 384 | **2.98 (2.50-3.55)** | <.05 | 560 | **1.91 (1.62-2.26)** | <.05 |
| Antidepressants with OCD or anxiety indication following initial diagnosis | No | 465 | 1.00 (ref) |  | 430 | 1.00 (ref) |  |
|  | Yes | 111 | **2.34 (1.89-2.89)** | <.05 | 342 | **1.75 (1.51-2.03)** | <.05 |
| Anxiolytics following initial diagnosis | No | 456 | 1.00 (ref) |  | 618 | 1.00 (ref) |  |
|  | Yes | 120 | **3.35 (2.71-4.13)** | <.05 | 154 | **2.69 (2.24-3.24)** | <.05 |
| Hypnotics and sedatives following initial diagnosis | No | 453 | 1.00 (ref) |  | 677 | 1.00 (ref) |  |
|  | Yes | 123 | **2.65 (2.15-3.28)** | <.05 | 95 | **2.16 (1.73-2.71)** | <.05 |
| Other medications following initial diagnosis | No | 426 | 1.00 (ref) |  | 513 | 1.00 (ref) |  |
|  | Yes | 150 | **2.96 (2.45-3.57)** | <.05 | 259 | **2.06 (1.77-2.39)** | <.05 |
| Any medications following initial diagnosis | No | 156 | 1.00 (ref) |  | 171 | 1.00 (ref) |  |
|  | Yes | 420 | **3.33 (2.76-4.02)** | <.05 | 601 | **2.20 (1.84-2.63)** | <.05 |
| Any medications with OCD or anxiety indication following initial diagnosis | No | 456 | 1.00 (ref) |  | 420 | 1.00 (ref) |  |
|  | Yes | 120 | **2.37 (1.93-2.91)** | <.05 | 352 | **1.81 (1.56-2.10)** | <.05 |
| **Non-significant predictors** | |  |  |  |  |  |  |
| Maternal unemployment | No | 477 | 1.00 (ref) |  | 614 | 1.00 (ref) |  |
|  | Yes | 99 | 1.22 (0.98-1.51) | .319 | 155 | 1.15 (0.96-1.38) | .975 |
| Paternal unemployment | No | 514 | 1.00 (ref) |  | 676 | 1.00 (ref) |  |
|  | Yes | 56 | 1.25 (0.95-1.65) | .319 | 85 | 1.12 (0.90-1.41) | .975 |
| Paternal income | Medium to high | 507 | 1.00 (ref) |  | 699 | 1.00 (ref) |  |
|  | Low | 63 | 1.25 (0.96-1.62) | .319 | 62 | 0.93 (0.71-1.21) | .975 |
| Multiple birth | Singleton | 544 | 1.00 (ref) |  | 752 | 1.00 (ref) |  |
|  | Twin, triplet or quadruplet | 32 | 0.62 (0.43-0.89) | .108 | 20 | 1.14 (0.73-1.78) | .975 |
| Gestational age | Preterm | 48 | 1.51 (1.12-2.04) | .078 | 49 | 0.96 (0.72-1.28) | .975 |
|  | Term | 484 | 1.00 (ref) |  | 658 | 1.00 (ref) |  |
|  | Post-term | 36 | 0.70 (0.50-0.98) | .319 | 55 | 0.82 (0.62-1.08) | .975 |
| Caesarean section | No | 526 | 1.00 (ref) |  | 703 | 1.00 (ref) |  |
|  | Yes | 50 | 1.42 (1.06-1.91) | .189 | 69 | 0.96 (0.75-1.24) | .975 |
| Smoking during pregnancy | No | 139 | 1.00 (ref) |  | 254 | 1.00 (ref) |  |
|  | Yes | 57 | 1.35 (0.99-1.84) | .319 | 97 | 1.03 (0.82-1.31) | .975 |
| Autoimmune diseases with brain-reactive antibodies | No | 560 | 1.00 (ref) |  | 757 | 1.00 (ref) |  |
|  | Yes | 16 | 1.43 (0.87-2.35) | .319 | 15 | 1.37 (0.82-2.28) | .975 |
| OCD in first degree relatives | No |  |  |  | 724 | 1.00 (ref) |  |
|  | Yes |  |  |  | 48 | 1.21 (0.90-1.62) | .975 |
| Any eating disorder in first degree relatives | No | 551 | 1.00 (ref) |  |  |  |  |
|  | Yes | 25 | 1.11 (0.74-1.65) | .625 |  |  |  |
| Other medications prior to initial diagnosis | No | 561 | 1.00 (ref) |  | 716 | 1.00 (ref) |  |
|  | Yes | 15 | 1.89 (1.13-3.16) | .175 | 56 | 1.27 (0.96-1.66) | .975 |

*Note*. Significant hazard ratios are bolded. Multiple testing was controlled for using the Benjamini-Hochberg procedure. AN = anorexia nervosa; OCD = obsessive-compulsive disorder; ref = reference level.

^a^ Although this predictor was also significant for subsequent AN, it was associated with *decreased* risk, opposite to the direction observed for subsequent anxiety disorder.

**Table 4**

*Hazard Ratios for Subsequent OCD Among Individuals with AN and Subsequent AN Among Individuals with OCD (Adjusted for History of Psychiatric Disorders)*

| Predictor | | Subsequent OCD among individuals with AN | | Subsequent AN among individuals with OCD | |
| --- | --- | --- | --- | --- | --- |
|  | | *HR* (95% CI) | Adjusted *p* | *HR* (95% CI) | Adjusted *p* |
| **Significant predictors unique to subsequent OCD or AN** | |  |  |  |  |
| Birth weight | <2500g | 0.85 (0.45-1.61) | .802 |  |  |
|  | 2500–4499g | 1.00 (ref) |  |  |  |
|  | ≥4500g | **3.06 (1.61-5.80)** | <.05 |  |  |
| Other eating disorders | No | 1.00 (ref) |  | 1.00 (ref) |  |
|  | Yes | 1.64 (1.20-2.25) | .064 | **7.19 (4.49-11.52)** | <.05 |
| **Significant predictors also associated with subsequent anxiety disorder** | |  |  |  |  |
| Other psychiatric disorders | No | 1.00 (ref) |  | 1.00 (ref) |  |
|  | Yes | **2.78 (1.93-4.01)** | <.05 | 0.93 (0.59-1.44) | .948 |
| Any anxiety disorder in first degree relatives^a^ | No | 1.00 (ref) |  | 1.00 (ref) |  |
|  | Yes | 1.18 (0.83-1.68) | .802 | **0.31 (0.17-0.58)** | <.05 |
| Any anxiety disorder in female first degree relatives^a^ | No | 1.00 (ref) |  | 1.00 (ref) |  |
|  | Yes | 1.20 (0.80-1.80) | .802 | **0.21 (0.09-0.52)** | <.05 |
| Antidepressants following initial diagnosis | No | 1.00 (ref) |  | 1.00 (ref) |  |
|  | Yes | **4.53 (3.20-6.41)** | <.05 | 1.59 (1.05-2.41) | .810 |
| Antidepressants with OCD or anxiety indication following initial diagnosis | No | 1.00 (ref) |  | 1.00 (ref) |  |
|  | Yes | **3.80 (2.72-5.30)** | <.05 | **1.88 (1.27-2.78)** | <.05 |
| Anxiolytics following initial diagnosis | No | 1.00 (ref) |  | 1.00 (ref) |  |
|  | Yes | **2.18 (1.48-3.22)** | <.05 | 1.65 (0.95-2.88) | .948 |
| Other medications following initial diagnosis | No | 1.00 (ref) |  | 1.00 (ref) |  |
|  | Yes | **1.96 (1.37-2.79)** | <.05 | 0.72 (0.43-1.21) | .948 |
| Any medications following initial diagnosis | No | 1.00 (ref) |  | 1.00 (ref) |  |
|  | Yes | **4.60 (3.18-6.64)** | <.05 | 1.47 (0.97-2.24) | .948 |
| Any medications with OCD or anxiety indication following initial diagnosis | No | 1.00 (ref) |  | 1.00 (ref) |  |
|  | Yes | **3.91 (2.82-5.42)** | <.05 | 1.83 (1.24-2.70) | .064 |
| **Non-significant predictors** | |  |  |  |  |
| Age at initial diagnosis (per year) | | 1.06 (1.01-1.12) | .768 | 1.03 (0.96-1.10) | .948 |
| Age at initial diagnosis (per *SD*) | | 1.27 (1.02-1.59) | .768 | 1.20 (0.79-1.83) | .948 |
| Maternal education | High school or above | 1.00 (ref) |  | 1.00 (ref) |  |
|  | Less than high school | 1.14 (0.84-1.56) | .802 | 0.67 (0.43-1.04) | .948 |
| Paternal education | High school or above | 1.00 (ref) |  | 1.00 (ref) |  |
|  | Less than high school | 1.09 (0.78-1.52) | .802 | 0.50 (0.29-0.85) | .340 |
| Maternal unemployment | No | 1.00 (ref) |  | 1.00 (ref) |  |
|  | Yes | 0.90 (0.59-1.35) | .802 | 0.98 (0.62-1.55) | .948 |
| Paternal unemployment | No | 1.00 (ref) |  | 1.00 (ref) |  |
|  | Yes | 0.83 (0.48-1.44) | .802 | 0.77 (0.41-1.45) | .948 |
| Maternal income | Medium to high | 1.00 (ref) |  | 1.00 (ref) |  |
|  | Low | 0.85 (0.51-1.42) | .802 | 0.94 (0.49-1.80) | .948 |
| Paternal income | Medium to high | 1.00 (ref) |  | 1.00 (ref) |  |
|  | Low | 0.80 (0.46-1.39) | .802 | 0.96 (0.49-1.91) | .948 |
| Multiple birth^a^ | Singleton |  |  |  |  |
|  | Twin, triplet or quadruplet |  |  |  |  |
| Gestational age | Preterm | 0.60 (0.28-1.28) | .802 | 0.57 (0.23-1.41) | .948 |
|  | Term | 1.00 (ref) |  | 1.00 (ref) |  |
|  | Post-term | 0.86 (0.50-1.49) | .802 | 0.79 (0.39-1.63) | .948 |
| Caesarean section | No | 1.00 (ref) |  | 1.00 (ref) |  |
|  | Yes | 1.22 (0.71-2.08) | .802 | 1.38 (0.81-2.38) | .948 |
| Smoking during pregnancy | No | 1.00 (ref) |  | 1.00 (ref) |  |
|  | Yes | 1.31 (0.77-2.21) | .802 | 0.59 (0.32-1.07) | .948 |
| Adversity score | 0 | 1.00 (ref) |  | 1.00 (ref) |  |
|  | 1 | 0.78 (0.54-1.13) | .802 | 0.78 (0.51-1.20) | .948 |
|  | 2 or more | 0.81 (0.40-1.65) | .802 | 0.62 (0.27-1.43) | .948 |
| Autoimmune diseases with brain-reactive antibodies | No | 1.00 (ref) |  |  |  |
|  | Yes | 2.41 (1.23-4.72) | .280 |  |  |
| Other autoimmune or autoinflammatory diseases | No | 1.00 (ref) |  | 1.00 (ref) |  |
|  | Yes | 1.16 (0.64-2.09) | .802 | 1.29 (0.56-2.95) | .948 |
| Any autoimmune or autoinflammatory disease | No | 1.00 (ref) |  | 1.00 (ref) |  |
|  | Yes | 1.62 (1.01-2.58) | .802 | 1.03 (0.45-2.36) | .948 |
| OCD in first degree relatives | No | 1.00 (ref) |  |  |  |
|  | Yes | 2.07 (0.92-4.69) | .802 |  |  |
| Any eating disorder in first degree relatives | No | 1.00 (ref) |  |  |  |
|  | Yes | 1.09 (0.54-2.23) | .802 |  |  |
| Any anxiety disorder in male first degree relatives | No | 1.00 (ref) |  | 1.00 (ref) |  |
|  | Yes | 1.26 (0.75-2.11) | .802 | 0.50 (0.22-1.13) | .948 |
| Any psychiatric disorder in first degree relatives | No | 1.00 (ref) |  | 1.00 (ref) |  |
|  | Yes | 1.16 (0.86-1.56) | .802 | 0.83 (0.56-1.21) | .948 |
| Antidepressants prior to initial diagnosis | No | 1.00 (ref) |  | 1.00 (ref) |  |
|  | Yes | 1.80 (1.19-2.70) | .150 | 1.40 (0.88-2.22) | .948 |
| Antidepressants with OCD or anxiety indication prior to initial diagnosis | No | 1.00 (ref) |  | 1.00 (ref) |  |
|  | Yes | 2.62 (1.20-5.71) | .405 | 1.47 (0.78-2.74) | .948 |
| Anxiolytics prior to initial diagnosis | No | 1.00 (ref) |  | 1.00 (ref) |  |
|  | Yes | 1.20 (0.56-2.60) | .802 | 1.22 (0.63-2.39) | .948 |
| Hypnotics and sedatives prior to initial diagnosis | No | 1.00 (ref) |  |  |  |
|  | Yes | 2.42 (1.24-4.73) | .280 |  |  |
| Other medications prior to initial diagnosis | No |  |  | 1.00 (ref) |  |
|  | Yes |  |  | 0.63 (0.25-1.60) | .948 |
| Any medications prior to initial diagnosis | No | 1.00 (ref) |  | 1.00 (ref) |  |
|  | Yes | 1.80 (1.23-2.64) | .093 | 1.13 (0.72-1.75) | .948 |
| Any medications with OCD or anxiety indication prior to initial diagnosis | No | 1.00 (ref) |  | 1.00 (ref) |  |
|  | Yes | 2.43 (1.11-5.30) | .675 | 1.39 (0.74-2.60) | .948 |
| Hypnotics and sedatives following initial diagnosis | No | 1.00 (ref) |  | 1.00 (ref) |  |
|  | Yes | 1.52 (1.01-2.29) | .802 | 1.57 (0.79-3.11) | .948 |

*Note*. Significant hazard ratios are bolded. Multiple testing was controlled for using the Benjamini-Hochberg procedure. AN = anorexia nervosa; OCD = obsessive-compulsive disorder; ref = reference level.

^a^ Although this predictor was also significant for subsequent anxiety disorder, it was associated with *increased* risk, opposite to the direction observed for subsequent AN.

^a^ Too few cases to report.

**Table 5**

*Hazard Ratios for Subsequent Anxiety Disorder Among Individuals with AN and OCD (Adjusted for History of Psychiatric Disorders)*

| Predictor | | Subsequent anxiety disorder among individuals with AN | | Subsequent anxiety disorder among individuals with OCD | |
| --- | --- | --- | --- | --- | --- |
|  | | *HR* (95% CI) | Adjusted *p* | *HR* (95% CI) | Adjusted *p* |
| **Significant predictors unique to subsequent anxiety disorder** | |  |  |  |  |
| Age at initial diagnosis (per year) | | 1.03 (1.00-1.06) | .428 | **1.06 (1.03-1.08)** | <.05 |
| Age at initial diagnosis (per *SD*) | | 1.12 (1.00-1.26) | .428 | **1.37 (1.21-1.56)** | <.05 |
| Maternal education | High school or above | 1.00 (ref) |  | 1.00 (ref) |  |
|  | Less than high school | **1.35 (1.13-1.60)** | <.05 | 1.20 (1.04-1.40) | .335 |
| Paternal education | High school or above | 1.00 (ref) |  | 1.00 (ref) |  |
|  | Less than high school | **1.35 (1.12-1.63)** | <.05 | 1.27 (1.08-1.49) | .071 |
| Maternal income | Medium to high | 1.00 (ref) |  | 1.00 (ref) |  |
|  | Low | **1.47 (1.16-1.88)** | <.05 | 1.00 (0.78-1.27) | .970 |
| Maternal unemployment | No | 1.00 (ref) |  | 1.00 (ref) |  |
|  | Yes | 1.20 (0.96-1.49) | .428 | 1.14 (0.95-1.36) | .970 |
| Paternal unemployment | No | 1.00 (ref) |  | 1.00 (ref) |  |
|  | Yes | 1.23 (0.93-1.62) | .428 | 1.11 (0.88-1.39) | .970 |
| Caesarean section | No | 1.00 (ref) |  | 1.00 (ref) |  |
|  | Yes | **1.40 (1.04-1.88)** | .349 | 0.96 (0.75-1.24) | .970 |
| Birth weight | <2500g | **1.73 (1.31-2.27)** | <.05 | 0.98 (0.73-1.32) | .970 |
|  | 2500–4499g | 1.00 (ref) |  | 1.00 (ref) |  |
|  | ≥4500g | 1.52 (0.91-2.55) | .428 | 0.96 (0.61-1.51) | .970 |
| Other autoimmune or autoinflammatory diseases | No | 1.00 (ref) |  | 1.00 (ref) |  |
|  | Yes | **1.60 (1.19-2.15)** | <.05 | 1.27 (0.91-1.77) | .970 |
| Any autoimmune or autoinflammatory disease | No | 1.00 (ref) |  | 1.00 (ref) |  |
|  | Yes | **1.56 (1.19-2.05)** | <.05 | 1.28 (0.95-1.72) | .970 |
| Other eating disorders | No | 1.00 (ref) |  |  |  |
|  | Yes | **1.48 (1.23-1.79)** | <.05 |  |  |
| Any anxiety disorder in male first degree relatives | No | 1.00 (ref) |  | 1.00 (ref) |  |
|  | Yes | 1.54 (1.16-2.04) | .063 | **1.41 (1.14-1.74)** | <.05 |
| Any psychiatric disorder in first degree relatives | No | 1.00 (ref) |  | 1.00 (ref) |  |
|  | Yes | **1.54 (1.30-1.81)** | <.05 | **1.42 (1.23-1.64)** | <.05 |
| Anxiolytics prior to initial diagnosis | No | 1.00 (ref) |  | 1.00 (ref) |  |
|  | Yes | **1.86 (1.28-2.71)** | <.05 | 1.41 (1.09-1.81) | .184 |
| Any medications prior to initial diagnosis | No | 1.00 (ref) |  | 1.00 (ref) |  |
|  | Yes | **1.63 (1.31-2.03)** | <.05 | 1.28 (1.09-1.51) | .071 |
| Hypnotics and sedatives following initial diagnosis | No | 1.00 (ref) |  | 1.00 (ref) |  |
|  | Yes | **2.54 (2.06-3.14)** | <.05 | **2.10 (1.67-2.62)** | <.05 |
| **Significant predictors also associated with subsequent OCD or AN** | |  |  |  |  |
| Other psychiatric disorders | No | 1.00 (ref) |  | 1.00 (ref) |  |
|  | Yes | **2.22 (1.82-2.70)** | <.05 | **1.78 (1.50-2.10)** | <.05 |
| Any anxiety disorder in first degree relatives^a^ | No | 1.00 (ref) |  | 1.00 (ref) |  |
|  | Yes | **1.49 (1.22-1.81)** | <.05 | **1.43 (1.22-1.67)** | <.05 |
| Any anxiety disorder in female first degree relatives^a^ | No | 1.00 (ref) |  | 1.00 (ref) |  |
|  | Yes | 1.36 (1.08-1.71) | .143 | **1.38 (1.16-1.65)** | <.05 |
| Antidepressants following initial diagnosis | No | 1.00 (ref) |  | 1.00 (ref) |  |
|  | Yes | **2.89 (2.42-3.45)** | <.05 | **1.88 (1.59-2.22)** | <.05 |
| Antidepressants with OCD or anxiety indication following initial diagnosis | No | 1.00 (ref) |  | 1.00 (ref) |  |
|  | Yes | **2.27 (1.84-2.80)** | <.05 | **1.73 (1.49-2.01)** | <.05 |
| Anxiolytics following initial diagnosis | No | 1.00 (ref) |  | 1.00 (ref) |  |
|  | Yes | **3.19 (2.59-3.95)** | <.05 | **2.65 (2.20-3.19)** | <.05 |
| Other medications following initial diagnosis | No | 1.00 (ref) |  | 1.00 (ref) |  |
|  | Yes | **2.80 (2.31-3.38)** | <.05 | **2.01 (1.72-2.35)** | <.05 |
| Any medications following initial diagnosis | No | 1.00 (ref) |  | 1.00 (ref) |  |
|  | Yes | **3.23 (2.67-3.90)** | <.05 | **2.15 (1.80-2.58)** | <.05 |
| Any medications with OCD or anxiety indication following initial diagnosis | No | 1.00 (ref) |  | 1.00 (ref) |  |
|  | Yes | **2.31 (1.88-2.84)** | <.05 | **1.80 (1.55-2.08)** | <.05 |
| **Non-significant predictors** | |  |  |  |  |
| Paternal income | Medium to high | 1.00 (ref) |  | 1.00 (ref) |  |
|  | Low | 1.23 (0.94-1.60) | .428 | 0.91 (0.70-1.19) | .970 |
| Multiple birth | Singleton | 1.00 (ref) |  | 1.00 (ref) |  |
|  | Twin, triplet or quadruplet | 0.63 (0.44-0.90) | .159 | 1.15 (0.73-1.79) | .970 |
| Gestational age | Preterm | 1.52 (1.13-2.04) | .114 | 0.94 (0.71-1.26) | .970 |
|  | Term | 1.00 (ref) |  | 1.00 (ref) |  |
|  | Post-term | 0.70 (0.50-0.98) | .428 | 0.81 (0.62-1.07) | .970 |
| Smoking during pregnancy | No | 1.00 (ref) |  | 1.00 (ref) |  |
|  | Yes | 1.34 (0.98-1.83) | .428 | 1.03 (0.81-1.30) | .970 |
| Adversity score | 0 | 1.00 (ref) |  | 1.00 (ref) |  |
|  | 1 | 1.15 (0.95-1.40) | .428 | 1.15 (0.98-1.35) | .970 |
|  | 2 or more | 1.62 (1.16-2.27) | .079 | 1.49 (1.15-1.94) | .071 |
| Autoimmune diseases with brain-reactive antibodies | No | 1.00 (ref) |  | 1.00 (ref) |  |
|  | Yes | 1.41 (0.85-2.32) | .428 | 1.34 (0.80-2.23) | .970 |
| OCD in first degree relatives | No |  |  | 1.00 (ref) |  |
|  | Yes |  |  | 1.19 (0.89-1.60) | .970 |
| Any eating disorder in first degree relatives | No | 1.00 (ref) |  |  |  |
|  | Yes | 1.08 (0.72-1.62) | .700 |  |  |
| Hypnotics and sedatives prior to initial diagnosis | No | 1.00 (ref) |  | 1.00 (ref) |  |
|  | Yes | 1.62 (1.04-2.54) | .428 | 1.56 (1.11-2.21) | .241 |
| Other medications prior to initial diagnosis | No | 1.00 (ref) |  | 1.00 (ref) |  |
|  | Yes | 1.40 (0.82-2.37) | .428 | 1.12 (0.84-1.49) | .970 |
| Any medications with OCD or anxiety indication prior to initial diagnosis | No | 1.00 (ref) |  | 1.00 (ref) |  |
|  | Yes | 1.91 (1.18-3.09) | .152 | 1.18 (0.92-1.51) | .970 |

*Note*. Significant hazard ratios are bolded. Multiple testing was controlled for using the Benjamini-Hochberg procedure. AN = anorexia nervosa; OCD = obsessive-compulsive disorder; ref = reference level.

^a^ Although this predictor was also significant for subsequent anxiety disorder, it was associated with *increased* risk, opposite to the direction observed for subsequent AN.
